# Alteration effects of karstification and hydrothermalism on middle Permian Qixia formation at the Wulong section, South China

**DOI:** 10.1038/s41598-023-40334-y

**Published:** 2023-08-12

**Authors:** Junxin Shang, Mingyou Feng, Xingzhi Wang, Benjian Zhang, Liang Xu, Xiaohong Liu

**Affiliations:** 1 School of Geoscience and Technology, Southwest Petroleum University, Chengdu, 610500 China; 2 Research Institute of Exploration and Development, PetroChina Southwest Oil and Gas Filed Company, Chengdu, 610041 China

**Keywords:** Geochemistry, Geology, Mineralogy, Petrology, Sedimentology

## Abstract

Middle Permian Qixia Formation in the southwestern region of Sichuan (SW China) has experienced multiphase fluidisation, resulting in an unclear understanding of the reservoir reconstruction effect. In this study, a systematic analysis of the Qi2 member in Wulong Town was carried out by combining field outcrop petrology and geochemistry. The results demonstrated that multiple sets of crystalline dolomite-bioclastic limestone cycles were stacked vertically in the Qi2 member, accompanied by the development of fractures and karst channels. The dolomite was mainly composed of silty-fine dolomite (D1) and recrystallised dolomite (D2). Furthermore, obvious multiphase dolomitic cements (Cd1-Cd2) were present in the fractures and pores. Early karst is known to have lithologic mutation surface development and karst channel development at the top of several secondary cycles. The vadose silt dolomites (Cd1) having karst channels developed dull luminescence under cathode luminescence (CL). Both the geochemical indicators of elements and rare earth element (REE) content indicated dysoxic-oxic environmental conditions. The hydrothermal solution displayed tectonic carniole characteristics in the strata burial stage. Fractures and pores were filled with hydrothermal minerals such as coarse dolomites-saddle dolomites (Cd2, with some caused by recrystallisation of the Cd1 hydrothermal solution) and fluorites. Coarse dolomites-saddle dolomites developed dull-red luminescence with a bright-red rim under CL and their δ^18^O_VPDB_ values were more negative than those of middle Permian limestone samples. Both the geochemical indicators of elements and REE content indicated the suboxic-anoxic environmental conditions. Karstification had minor constructive impact on the reservoir of the Qi2 member in Baoxing in southwestern Sichuan. Most products of karstification were distributed as fillings in channels. Aside from creating certain networked fractures, the hydrothermal solution was mainly filled with hydrothermal minerals along the fractures, pores and early karst channels. Karst and the hydrothermal solution mainly damaged the middle and upper parts of the middle Permian Qixia Formation in Southwest Sichuan. The impact of episodic fluid on the restoration of the carbonate reservoir was mainly restricted by channels for fluid migration and thickness differences among the reservoir. However, certain thick-layered and massive crystalline dolomite may hold promise for exploration.

## Introduction

Tectonic and sea level changes may cause extensive fluid migration^1^ and karstification in many sedimentary basins. This fluid can be composed of evaporated brine, seawater and hydrothermal solutions^2–4^, which may induce the dolomitisation of carbonate formations under specific physical and chemical conditions^5–7^. In the field of petroleum geology, considerable attention has been paid to the dolomite reservoirs produced by hydrothermal solutions, which are a common type of dolomitisation fluid^8,9^. Additionally, carbonatite dissolution (karstification) by surface unsaturated fluids may also produce karst-type reservoirs that are of significance to the development of carbonate reservoirs^10–12^. Karst reservoirs account for 20–30% of explored carbonatite petroleum and natural gas reservoirs^13^. They are extensively distributed globally, including South America^14^, Australia^15^ and the Sichuan Basin in China^16^. However, certain carbonatites went through restoration by the simultaneous karstification of unsaturated fluid and alteration of middle-deep buried fluids^17,18^. The collective impact of karstification and alteration on reservoirs still requires further investigation.

The middle Permian dolomite reservoir in Sichuan Basin (SW, China) is an important natural gas exploration series. It has attracted considerable research attention because of its special structural framework, complicated sedimentary environment and reservoir-forming conditions^19,20^. The formation of middle Permian dolomite reservoirs is mainly related to dolomitisation. However, the reservoir performance is constrained by various factors such as the sedimentary phase, karstification and tectonism. The degree to which the bank facies sedimentation superimposed on the eogenetic karst is preserved during the burial process remains ambiguous. Moreover, the controversy persists regarding whether organic acids or thermal fluids are responsible for large-scale reservoir reconstruction in areas with complex tectonic superposition and medium-deep burial conditions. It has been found that strata having karst systems have good potential for exploration and development^21,22^. However, it remains uncertain whether the modification effect is beneficial to reservoir development during the combined influence of late multi-phase tectonic/fluidic events on local strata.

Following the hydrothermal dolomitisation and initial in-depth studies, numerous studies have been conducted on reservoir modification by hydrothermal fluids^23–25^. However, different opinions could be found on the combined effects of thermal fluid on carbonate transformation. It has been reported that the rising thermal fluid controls the formation of high-porosity and high-permeability belts in fold or fault carbonate reservoirs via the strike-slip faults as channels^26,27^. Thermal fluid could block existing pores during migration, since CO_2_-rich thermal fluid may corrode surrounding rocks to form pores and precipitate saddle dolomite under specific conditions^28^. Hydrothermalism is a common phenomenon that has been observed in carbonate reservoirs in areas such as the Sichuan Basin^29^ and Bohai Basin^30^. The fluorine-containing thermal fluid can be a major factor that controls carbonate reservoir transformation^31^ and has established the paleokarst reservoir transformation pattern based on thermal fluid. This pattern summarises the process in which karstification provides fundamental pore frameworks for carbonate reservoir and thermal fluid reconstruction of the built reservoir space in the middle-deep burial stage^32^. In general, the debate is on the degree of dissolution-filling of pores and the improvement of the percolation capacity after the reconstruction of early pores by late fluids.

Middle Permian carbonate reservoirs in the southwestern region of Sichuan are commonly reformed by complex diagenetic fluids. What are the action mechanisms of such diagenetic fluids? What are their transformation effects? What are the characteristics of their spatial and temporal coupling? Therefore, a series of studies were carried out on the rock-geochemical characteristics in the middle Permian Qixia Formation (Qi2 member) of Baoxing and the southwestern region of Sichuan. The objective of this study was to (1) determine the lithological and geochemical characteristics of the Qixia Formation; (2) implement the diagenetic evolutionary process and (3) understand the combined impact of karst-thermal fluids on carbonate formation. The findings of this research could support oil and gas exploration in middle Permian carbonate reservoirs in the Sichuan Basin.

## Geological setting

**Figure 1:**
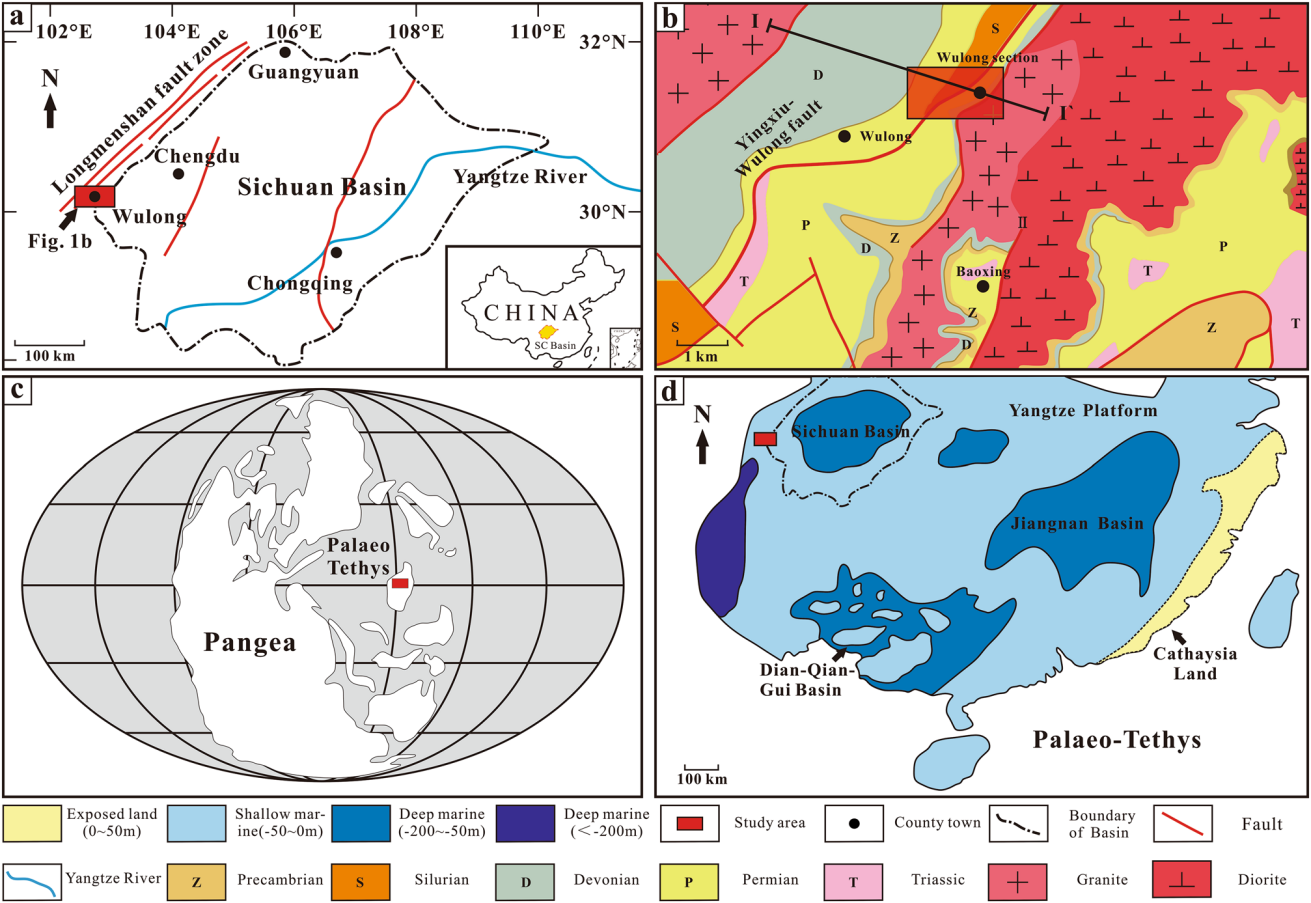
Geographic position of the Sichuan Basin and lithofacies palaeogeography. (**a**) Geographic position of the Sichuan Basin. (**b**) Geographic position of the study area. (**c**), (**d**) Lithofacies palaeogeography map during the Chihsian in South China and the location of the studied section (modified from Wang and Jin^34^).

**Figure 2:**
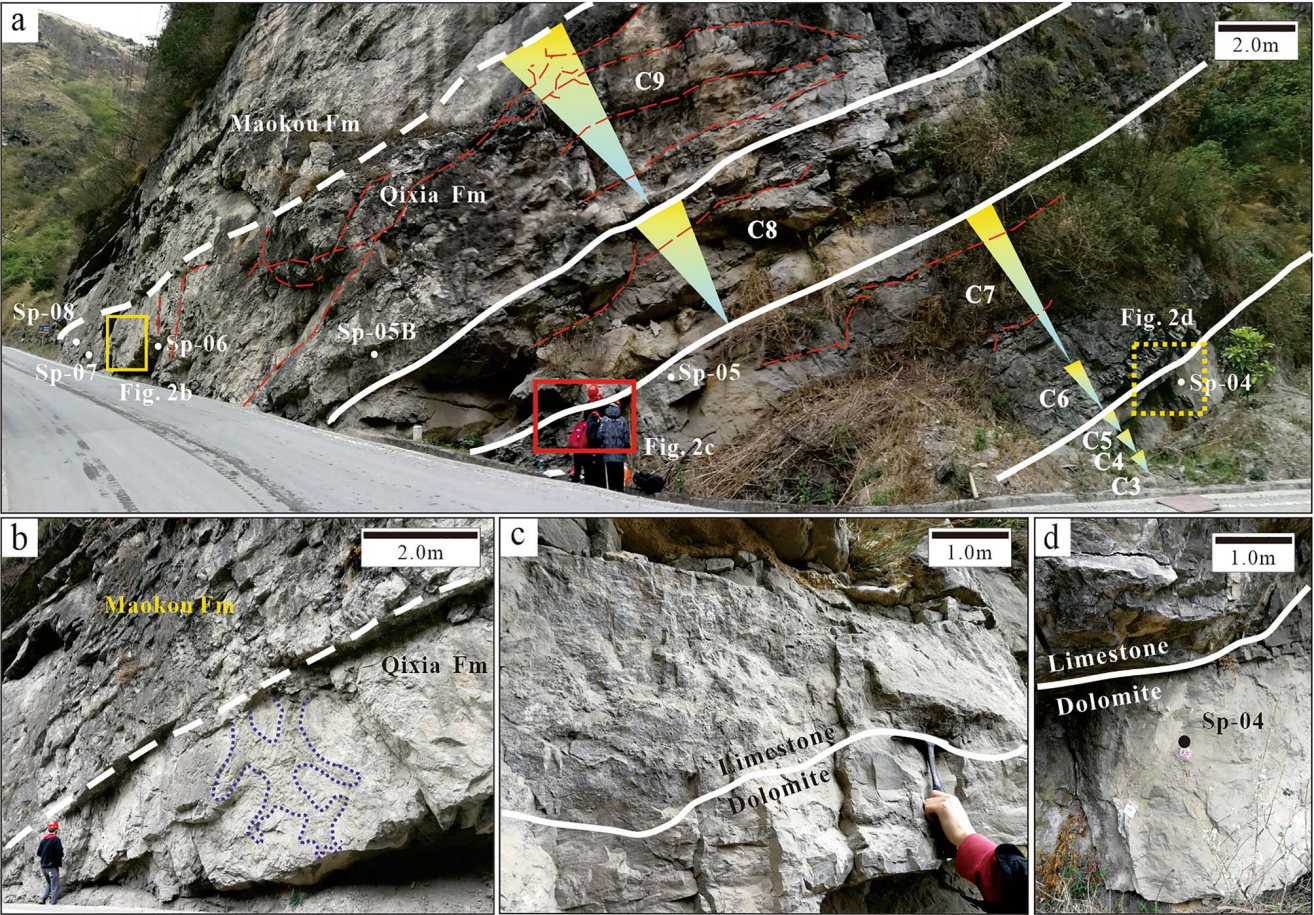
Field photos of the Second member, Qixia Formation, Wulong section and Southwest Sichuan Basin. (**a**) Division of the top Qi2 member into 7 cycles (C3–C9). The yellow inverted triangles denote regression. The red dotted lines denote fractures and the white solid lines are boundary lines of the sedimentary cycle. The white dotted line is the plane of unconformity. The yellow dotted line area is enlarged in Figure b. The red solid line area is enlarged in Figure c. The yellow solid line area is enlarged in Figure d. The white dots are sampling points. (**b**) The white dotted line refers to the plane of unconformity between the Qixia and Maokou Formations. The blue dotted line shows the karst channel. (**c**) The white solid line is the boundary line between C6 and C7, where the upper is limestone and the lower is dolomite. (**d**) The white solid line is the boundary line between C5 and C6, where the upper is limestone and the lower is dolomite.

The middle Permian Qixia Formation of the Wulong Section is situated in proximity to Wulong County in the southwestern region of the Sichuan Basin in southern China. From a tectonic perspective, it is very close to the Yingxiu-Wulong Fault (Figs. 1a,b and 2b) and is accompanied by the extensive occurrence of faults^33^. In terms of palaeogeography, the middle Permian Qixia Formation is a component of the north-western region of the Yangtze Platform and is close to the western edge of the Sichuan Basin. The Yangtze Platform was flooded due to transgression during middle Permian, resulting in its transformation into a shallow platform (Fig. 1c,d). According to previous studies, the overall water depth in the Yangtze Platform is about 50 m and could reach 200 m in certain deep-water areas^34^. The Longmenshan Fault Zone has undergone two major stages of tectonic evolution since the Longmen Mountains at the western edge of the Sichuan Basin formed a uniform base due to the influence of the Jinning Movement and Chengjiang Movement in the Proterozoic era. These stages are the tensional stage, from the Sinian to the Middle Triassic, and the extrusion stage, since the Late Triassic^35,36^. There has been strong rifting activity from the Late Silurian to the Middle Triassic, which reached its highest point during the Late Permian and was accompanied by large-scale magmation. This strong Permian rifting activity was named the ‘Emei Taphrogenesis’ ^37^. The Yingxiu-Wulong Fault is a component of the Longmenshan Central Fault. Subsequently, the Indosinian Movement, Yanshan Movement, Himalayan Movement and other tectonic-related events occurred. The strata of Qixia Formation were possibly reformed by thermal fluid-karst due to frequent changes in sea level and tectonic-thermal events.

Middle Permian strata of the Sichuan Basin are unconformable to the underlying Carboniferous strata after exposure for a long time. They are composed of the bottom Liangshan, middle Qixia and overlying Maokou Formation. The Liangshan Formation covers the regional plane of unconformity. It mainly consists of carbonaceous shale, coal and sandstone, which reflects the coastal marsh environment. The Qixia Formation consists of a complete tertiary cycle of transgression and regression, which can be divided into two members. The Qi1 member is mainly composed of limestone that is rich in organic matter with flint lumps at the bottom. It is classified as part of the transgression system tract. The Qi2 member is mainly composed of light grey-off-white crystalline dolomite with light grey micrite-sparry bioclastic limestone in local areas. It is part of the highstand systems tract and generally belongs to the open platform sedimentary environment. In this study, the main focus was on the upper parts (~ 20 m thick) of the Qi2 member, which was the key section for oil and gas exploration in the region. The telogenetic karst of the Qixia Formation underwent partial alteration after sedimentation, following which the sea level rose sharply and the Maokou Formation was developed. The Maokou Formation mainly had thin layers of dark grey-cinereus argillaceous limestone containing organic matter, with the common development of a nodular structure (Fig. 2a). It belongs to the low-energy deep-water sedimentary environment.

In the strata sequence, the vertical lithofacies variation of the ~ 40 m thick section in the Qi2 member showed clear cyclicity (Fig. 2a). The basic stratigraphic unit (cycle) was composed of two parts: the lower bioclastic limestone and the upper crystalline dolomite. This formed a meter-level upward shallow cycle. Such stratigraphic cyclicity is caused by short-term sea-level fluctuations^38^. In this study, a total of nine cycles (C1–C9) were recognised, among which C3–C9 were key research objects.

## Samples and methods

A total of 32 rock samples were collected successively from the Qi2 member of the Wulong Section for sedimentological and geochemical studies. Alizarin red stain was prepared to distinguish calcite and dolomite. One hundred thin sections from 32 rock samples were processed by double-side polishing and immersion in blue epoxy resin to determine their lithological composition, porosity and diagenetic sequence. Images of the processed thin sections were captured under an optical microscope (Olympus BX53M) and cathode luminescence microscope (CL8200MK5) at Southwest Petroleum University (Figs. 4 and 5). Trace elements and rare earth elements (14 samples) were analysed by Analytical Chemistry and Testing Services (ALS) in Guangzhou. Quantitative analyses were performed using lithium borate fusion and inductively coupled plasma mass spectrometry. Carbon and oxygen isotope (14 samples) tests were performed following the microdrill sampling at Southwest Petroleum University. These samples were prepared by the phosphate method and analysed using an IsoPrimeGC5 isotope mass spectrometer (Elementar, Germany). Stable isotope data were converted to permil (‰) relative to Vienna Pee Dee Belemnite (V-PDB) and corrected by fractionation factors mentioned by Fairchild and Spiro^39^. The precision of the δ^18^O and δ^13^C ratio data was better than ± 0.1‰. The rare earth element (REE) data were normalised to post-Archean Australian shale (PAAS)^40^. The REE anomalies were calculated by the classical equation^41^: δCe = Ce_N_ / (Pr_N_^2^/Nd_N_), δEu = 2Eu_N_/(Sm_N_ + Gd_N_).

## Petrography

**Figure 3:**
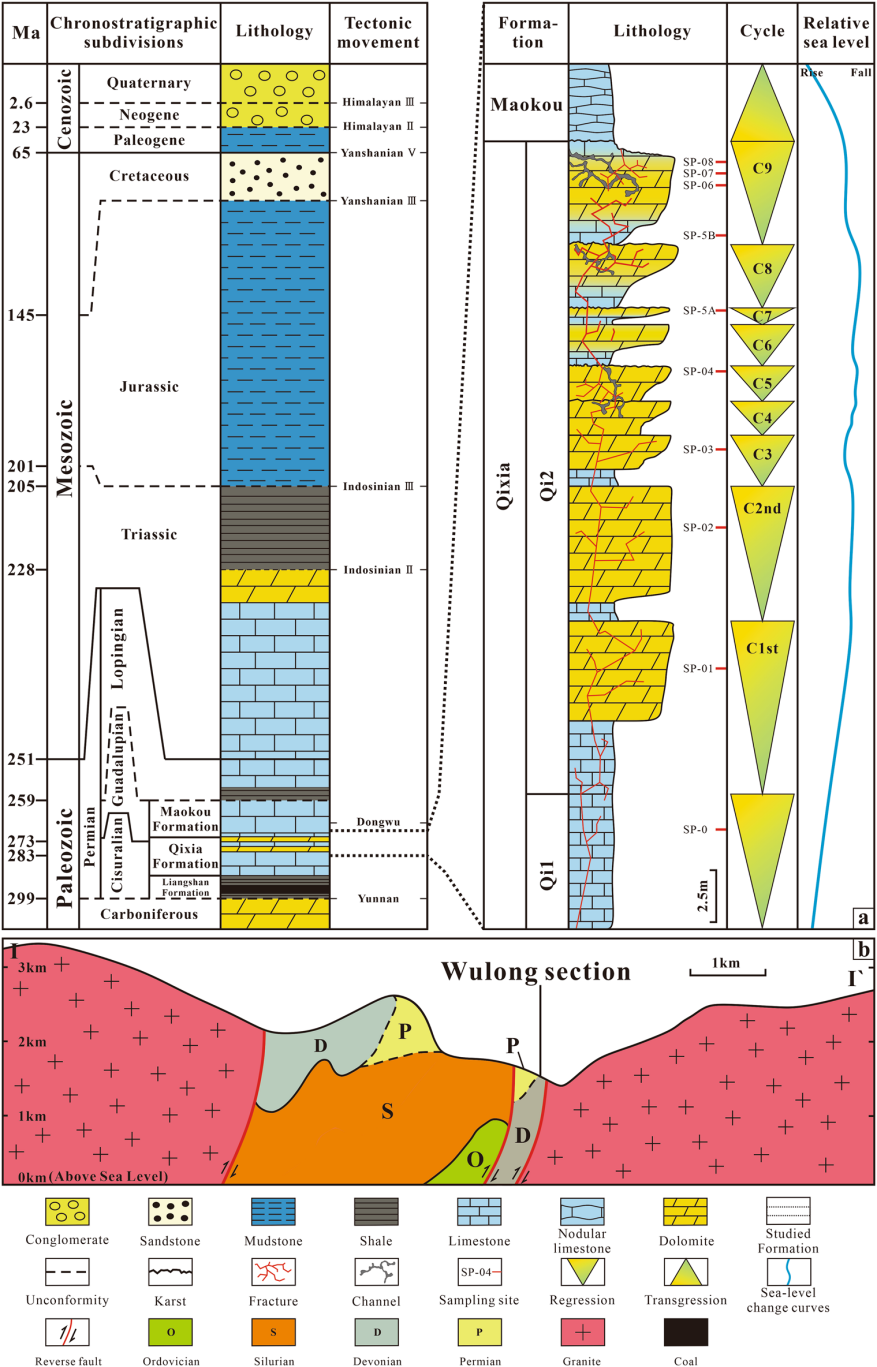
Lithological column and geological cross-section map. (**a**) Lithological column of Qixia Formation at the Wulong section. (**b**) Geological cross-section map of the Wulong section. Section line I–I’ corresponds to Fig. 1b.

In the Wulong Section close to the Yingxiu-Wulong Fault, the upper outcrop and the thin sections of the Qi2 member samples showed different lithological and structural features of carbonate rocks. The matrix limestone was found to be mainly grey-light grey thin-middle bioclastic limestone (L1). The thin–middle limestone and dolomite gradually came into contact along the vertical direction. The thick-massive dolomite mainly had abrupt vertical contact with limestone (Fig. 3a). The dolomite could be divided into three types, i.e., powder to fine dolostone (D1), recrystallised dolostone (D2) and dolomite cement (Cd). Additionally, calcite cement (CC), pyrite (Py) and fluorite (Fl) were present in residual fractures and pores. The types of lithofacies and petrologic characteristics are described below.

### Bioclastic limestone (L1)

**Figure 4:**
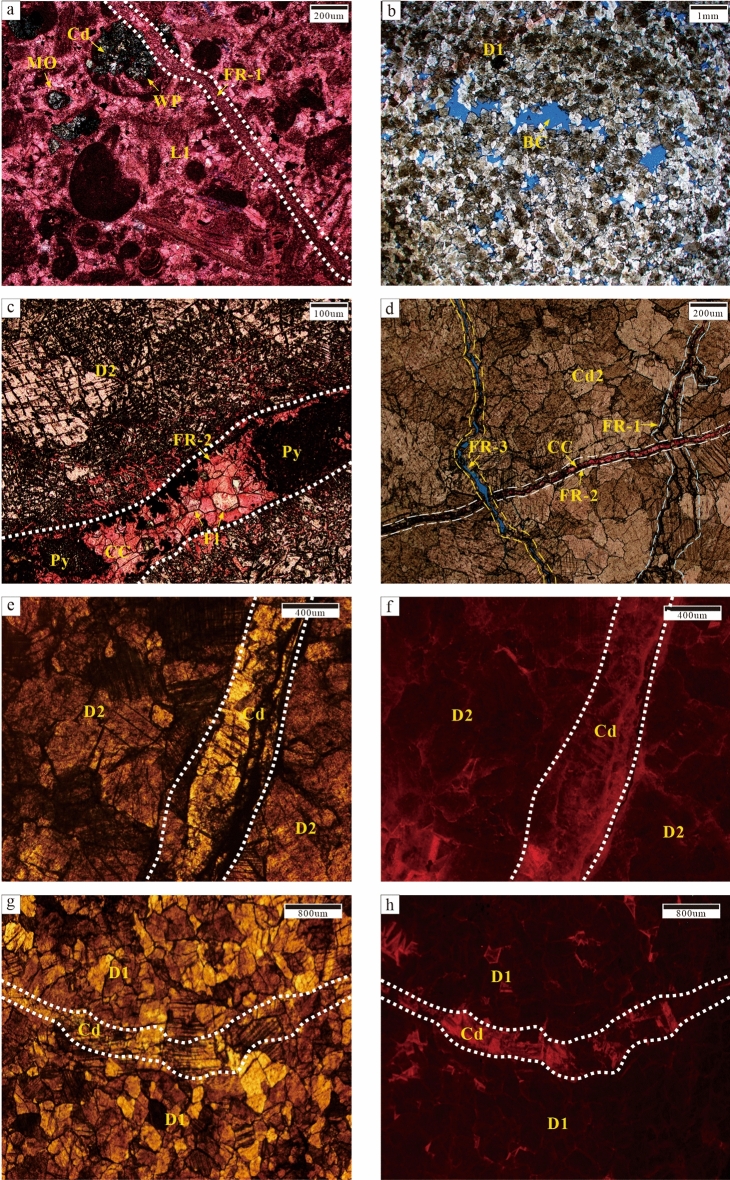
Petrographic observations of the Second Member, Qixia Formation and Wulong section. (**a**) Bioclastic limestone (L1), moldic porosity (MO) and intraparticle porosity are filled with dolomite cement (Cd). The white dotted line represents Phase-I fracture (FR-1) filled with CC and alizarin red staining (Sp-5B, C9). (**b**) Fine dolomite (D1) is composed of anhedral and subhedral crystals with residual particle structure; intercrystal porosity (BC) developed and porosity was 8% (Sp-06, C9). (**c**) Medium dolomite (D2), obvious brecciation. The white dotted line represents Phase-II fracture (FR-2) filled with Py, Fl and CC (Sp-5A, C7). (**d**) The blue dotted line represents FR-1 and the white dotted line represents FR-2. The yellow dotted line represents Phase-III fracture (FR-3) which cuts across FR-2, Cd2 and CC while FR-2 cuts FR-1 (Sp-5A, C7). (**e**) Medium to coarse dolomite (D2). The white dotted line represents fractured plane light (Sp-07, C9). (**f**) Figure e displays dull-red luminescence under CL. The white dotted line represents the fracture (Sp-07, C9). (**g**) Fine dolomite (D1). The white dotted line represents the fracture plane light (Sp-07, C9). (**h**) Figure g displays the dull luminescence under CL. The white dotted line represents the fracture (Sp-07, C9).

Bioclastic limestone was mainly formed at the upper part of the Qi2 member. It generally consisted of thin-middle strata and had a basic cycle with the overlying thick-massive crystalline dolomite strata. Numerous bioclastic particles were found to be present. Moldic and intragranular pores were completely filled by calcite. Whereas, some of them were dolomitised (vadose silt dolomite) and cut by late fractures (Fig. 4a).

### Dolostone (D1–D2)

Powder and fine dolostone (D1) were mainly formed at the fractures at the very top of Qi2. D1 was primarily composed of planar euhedral and subhedral crystals (0.1–0.25 mm), with residual bioclastic particles in local areas. Intercrystal pores in thick-massive (> 2 m thick) dolomite strata were well preserved and the porosity reached 8% (Fig. 4b). Some D1 might have further recrystallised into D2. Under cathode luminescence (CL), D1 displayed very dull luminescence while crystals near fractures showed bright-red rims (Fig. 4g,h).

Most of the recrystallised dolostone (D2) was formed near fractures (or microfractures). Crystals were found to be mainly non-planar anhedral (0.25–2 mm). D2 was formed inside the thin (< 2 m thick) dolomite strata while primarily having dense and unfilled fractures. The porosity was 1% (Fig. 4c,d). D2 was found to be a part of the recrystallisation of D1. Under CL, D2 showed dull red luminescence (Fig. 4f).

### Dolomite cement (Cd)

**Figure 5:**
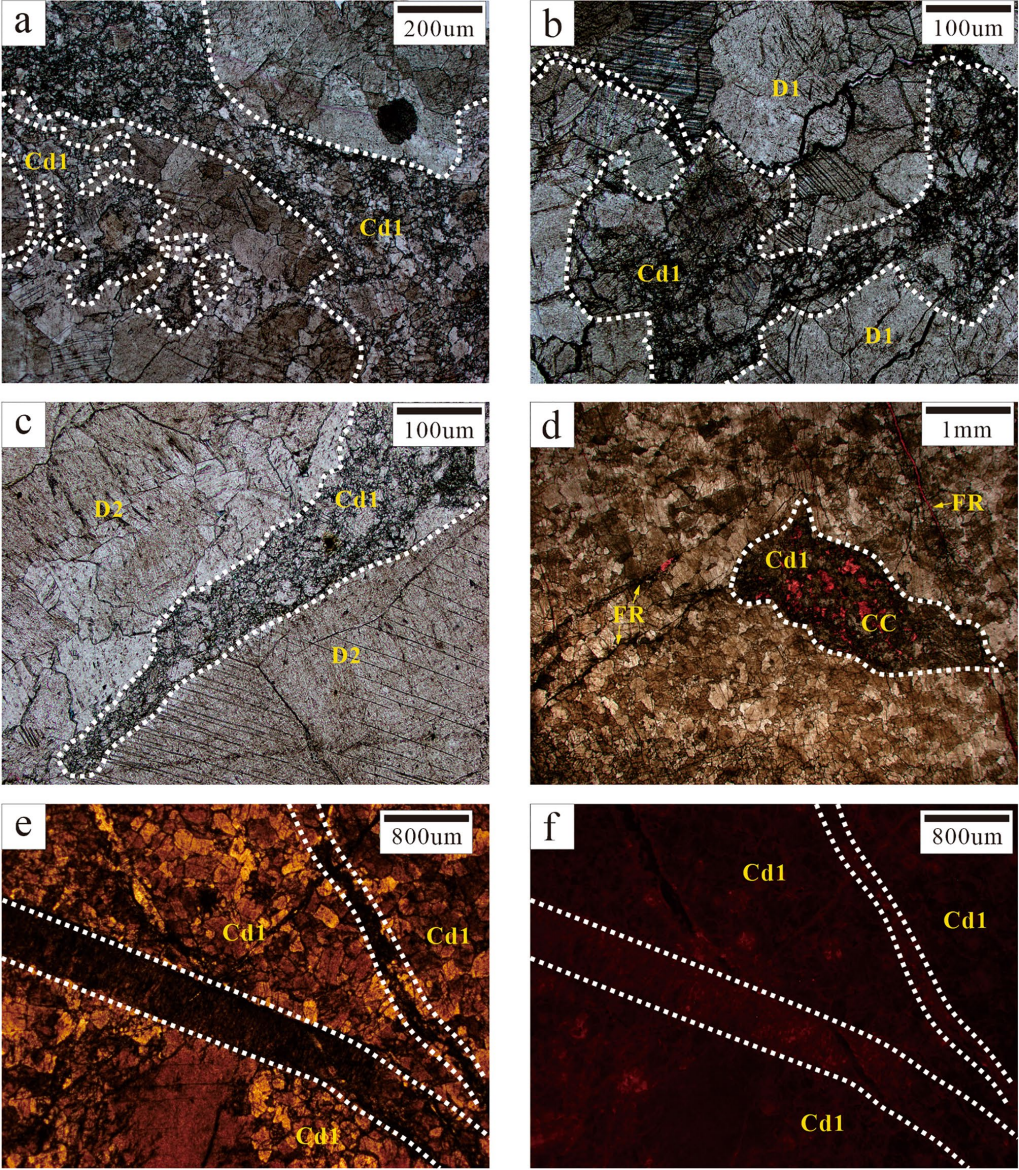
Karstification characteristics. (**a**, **b**, **c**) Karst channels (white dotted lines) are filled with Cd1 (Sp-04, C5; Sp-06, C9; Sp-07, C9). (**d**) Karst channels (white dotted lines) are filled with Cd1 and CC and connected by fractures (FR, yellow arrows); alizarin red staining (Sp-07, C9); (**e**, **f**) Cd1 on both sides of the fractures (white dotted lines) displayed dull luminescence under CL (Sp-04, C5).

Dolomite cement only appeared as pore-filling minerals. Cd could be further divided into Cd1 (vadose silt dolomite) and Cd2 (saddle dolomite and transitional form) according to its genesis. Cd1 was mainly composed of subhedral and euhedral crystals (0.05–0.25 mm) and was mainly formed in karst channels. Cd1 silt dolomite might have been carried by fluid media which passed through karst channels. It was not reformed or slightly reformed to a relatively small degree by late diagenetic fluids. Thus, it was speculated that Cd1 was the result of karstification (Fig. 5). Cd2 was mainly composed of anhedral and euhedral crystals (0.25–2 mm). Some crystals showed undulatory extinction under orthogonal luminescence and were found to be saddle-shaped. They were mainly filled with fractures and karst channels. Residual pores were filled with calcite cement (CC). Most Cd2 was formed through the further recrystallisation of Cd1 or D2, with only a portion of Cd2 being formed by direct precipitation in diagenetic fluid. It was speculated that Cd2 was the result of hydrothermalism (Fig. 4d).

### Hydrothermal minerals

Pyrite (Py) and fluorite (Fl) were precipitated after D2 and Cd. They represent the final diagenesis stage in the diagenetic sequence (Figs. 4c and 5d).

## Alteration of karstification and hydrothermalism characteristics

### Characteristics and spatial distribution of dolomite

Strata in the Qi2 member of the Wulong Section were about 40 m thick. In this study, the focus was given to the upper part of the Qi2 member (20 m), which could be divided into seven fourth-order cycles (C3–C9). Except for C4 and C5, each cycle was composed of the underlying thin bioclastic limestone layer and the overlying middle crystalline dolomite layer. C4 and C5 were sequenced with variable dolomite grain sizes (Fig. 3a). With changes in the sedimentary cycle, limestone and dolomite strata were formed upward in an alternating manner. Karst channels were formed at the top of most cycles (C4, C5, C7, C8 and C9) in the dolomite strata, with fractures in local areas. There were abundant residual bioclastic particles in the limestone strata, mainly including *Gastropoda, Barchiopoda* and *Echinodermata* (Fig. 4a).

### Characteristics of karstification

In contrast to telogenetic karst, the formation of eogenetic karst is based on the conduction media of karst water and different karst materials^42^. This karstification mode is driven by high-frequency fluctuations in sea levels while taking place around the penecontemporaneous stage^43^. Most contemporaneous sediments have high pore permeability and can provide channels for the diffusion of karst water. The diffusion of karst water among particles in the early stage resulted in the gradual formation of channels in local areas. Hence, double-porosity systems formed, combining channel porosity and interparticle porosity^42^. The characteristics of both telogenetic karst and eogenetic karst were found in the upper cycle of Qi2 member.

Karst characteristics were mainly evident in vertical lithologic sequence characteristics, lithologic fabric characteristics and pore characteristics. The lithologic sequence contained lithologic mutation surfaces, which are generally an important characteristic of eogenetic karst^44^. Lithologic mutation surfaces were found from C5 to C6 and from C7 to C8 in field outcrops. Specifically, the underlying bioclastic limestone strata changed abruptly into crystalline dolomite strata while having obvious mutation interfaces (Fig. 2a,b,c). The cycle top belonged to a high-frequency exposed surface. Many karst channels (0.1–0.3 mm) were found in outcrop and thin sections of the sample. They were mainly distributed at the top of cycles with harbour-shaped edges and internal spaces filled with Cd1 (Fig. 5a,b,c,d). Karst channels were found to be solution-enlarged matrix fractures with interparticle pores. They had a comprehensive manifestation of eogenetic karst and telogenetic karst characteristics^45^. The thin sections of the sample had considerable moldic and intraparticle porosity. This reflected selective corrosion and the dissolution of unstable aragonite in eogenetic bioclastic particles. Under CL, Cd1 in karst channels displayed dull luminescence (Fig. 5e,f).

### Characteristics of hydrothermalism

It is generally believed that hydrothermal dolomite reservoirs are formed by the reconstruction of precursor pores and the alteration of matrix limestone by the Mg-rich fluid, which is transported from the underlying strata to the overlying limestone strata through the fault system. This process has been verified in the Michigan Basin in the northeastern United States and the Appalachian Basin in eastern Canada^7,46,47^. Therefore, hydrothermalism characteristics are mainly recognised according to the formation of hydrothermal dolomite (HTD) and the dissolution of limestone.

**Figure 6:**
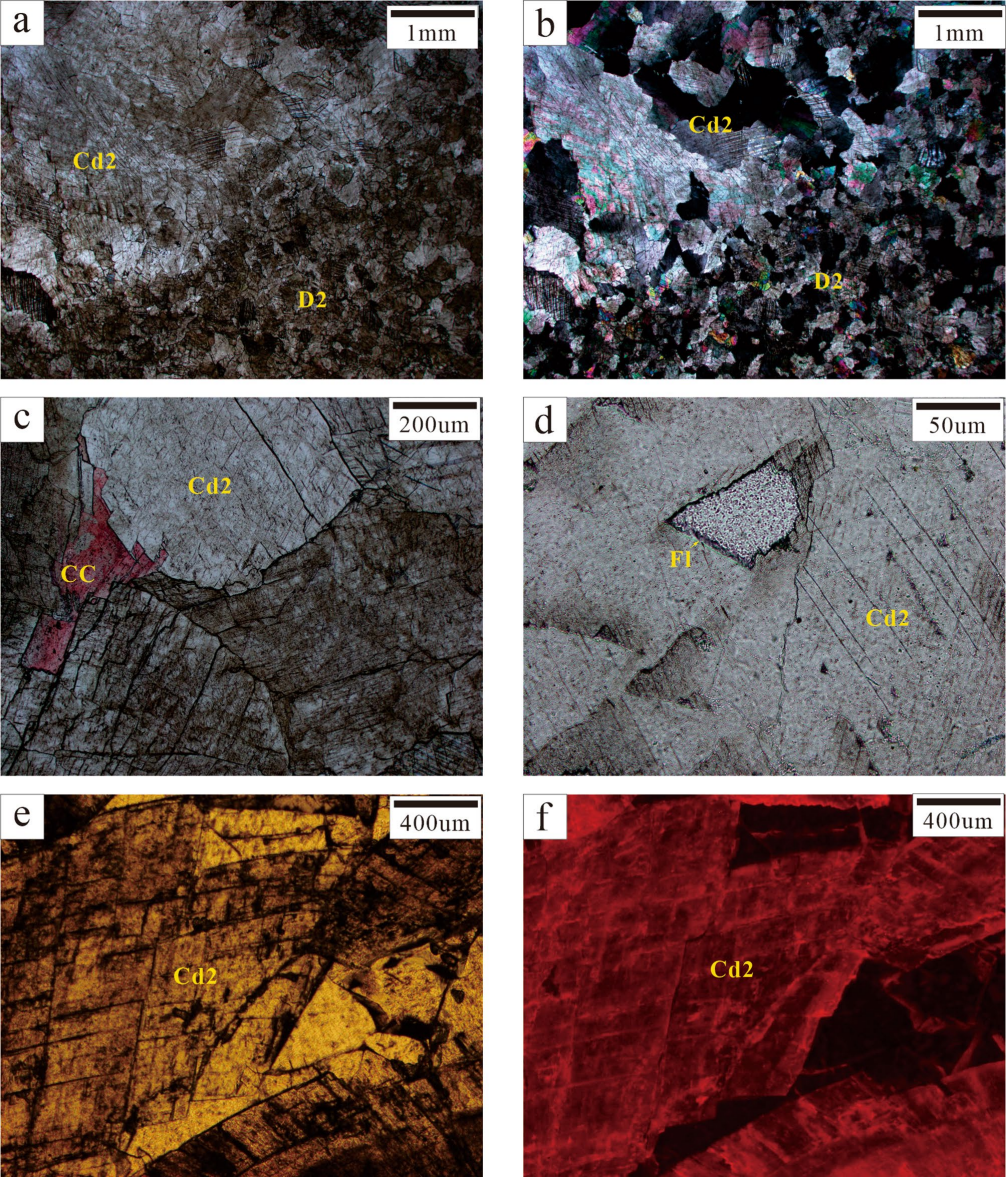
Hydrothermalism characteristics. (**a**, **b**, **c**) Saddle dolomite (Cd2) is developed near D2 and fractures are filled with CC. Saddle dolomite displayed undulatory extinction under orthogonal polarisation Figure b. Figure c used alizarin red for staining (Sp-06, C9; Sp-08, C9). (**d**) Fl is filled with residual pores (Sp-04, C5). (**e**, **f**) Pores are filled with saddle dolomite, which showed dull-red luminescence with a bright-red rim under CL (Sp-04, C5).

Hydrothermalism characteristics in a region are mainly manifested by petrofabrics and mineral combinations. Observation of the thin sections under a microscope revealed D2 to have obvious crystal brecciation (Fig. 4c). The dolomite crystals on both sides of the karst channel were broken in an interconnected pattern. The size and orientation of the crystals had controlling effect on the fragmentation of the petrofabrics and hence, resulting in directional fragmentation. This fragmentation may have been caused by hydraulic fracturing induced by high pressure generated during the hydrothermal fluid migration. The filling of pores with saddle dolomite (Cd2) was the most direct evidence of a hydrothermal environment^7^ (Fig. 6a,b,c). Other typical hydrothermal-associated materials such as Py and Fl. Py mainly existed in blocks and was distributed in some fractures (Fig. 4c). Fl was distributed in fractures and among breccia while having irregular crystal morphology and triangular solution formation (Fig. 6d). Under CL, Cd2 showed dull-red luminescence with a bright-red rim (Fig. 6e,f).

## Geochemistry

### Trace elements

According to trace elements data, the V/Cr and Ni/Co values of L1 in the Qi2 member were 0.5 and 0.29, respectively. The V/Cr value of D1 ranged from 0.12 to 1.2 and the Ni/Co value from 2.4 to 6.41. The V/Cr and Ni/Co values of medium-coarse crystalline dolomite (D2) ranged from 4–5.71 to 16.33–18.85, respectively. The V/Cr and Ni/Co values of Cd1 in Cd ranged from 0.33–1.75 to 4.1–7.82, respectively. The V/Cr and Ni/Co values of Cd2 were 4.5–5.82 and 11.76–14.1, respectively. The V/Cr and Ni/Co values of CC were 6 and 11.11, respectively (Table 1).

**Table 1:** Stable isotope and Trace element data of sample rocks in middle Permian in SW Sichuan Basin of WL-Wulong section.

Outcrop	Sample Identification			Stable Isotopes / (‰)		Trace elements / (μg/g)					
	Strata	Sample ID	Lithology	δ^13^C_VPDB_	δ^18^O_VPDB_	V	Ni	Cr	Co	V/Cr	Ni/Co
WL	Second member of Qixia Fm	Sp-5B	L1	3.44	− 1.20	4.00	0.40	8.00	1.40	0.50	0.29
WL	Second member of Qixia Fm	Sp-06-1	D1	2.15	− 2.30	4.00	3.59	32.30	0.56	0.12	6.41
WL	Second member of Qixia Fm	Sp-06-3	D1	2.31	− 2.90	4.40	0.84	10.30	0.35	0.43	2.40
WL	Second member of Qixia Fm	Sp-06-2	Cd1	1.96	− 1.90	3.30	0.61	9.90	0.08	0.33	7.82
WL	Second member of Qixia Fm	Sp-04-1	Cd2	2.78	− 9.83	2.10	0.80	0.40	0.07	5.25	11.76
WL	Second member of Qixia Fm	Sp-5A-1	D2	2.11	− 13.18	1.40	1.96	0.30	0.12	4.67	16.33
WL	Second member of Qixia Fm	Sp-07	D2	0.99	− 9.18	1.60	0.98	0.40	0.05	4.00	18.85
WL	Second member of Qixia Fm	Sp-08-1	CC	− 7.27	− 11.31	6.00	0.20	1.00	0.02	6.00	11.11
WL	Second member of Qixia Fm	Sp-04-2	D1	0.84	− 1.98	18.00	1.84	15.00	0.40	1.20	4.60
WL	Second member of Qixia Fm	Sp-04-5a	Cd1	3.28	− 2.20	15.75	0.30	9.00	0.06	1.75	5.00
WL	Second member of Qixia Fm	Sp-04-5b	Cd1	1.49	− 3.80	28.80	1.64	18.00	0.40	1.60	4.10
WL	Second member of Qixia Fm	Sp-04-2	Cd2	2.51	− 10.86	0.90	0.66	0.20	0.05	4.50	13.20
WL	Second member of Qixia Fm	Sp-5A-2	D2	1.56	− 11.36	2.86	1.43	0.50	0.08	5.71	17.85
WL	Second member of Qixia Fm	Sp-04-3	Cd2	2.70	− 11.15	3.49	0.85	0.60	0.06	5.82	14.10

V/Cr and Ni/Co values are often used to evaluate the redox environment of sedimentary water bodies during the formation of minerals^48^. The V/Cr and Ni/Co values of L1, D1 and Cd1 revealed dysoxic-oxic environmental conditions while the V/Cr and Ni/Co values of D2, Cd2 and CC revealed suboxic-anoxic environmental conditions (Fig. 8a).

### Rare earth elements

**Figure 7:**
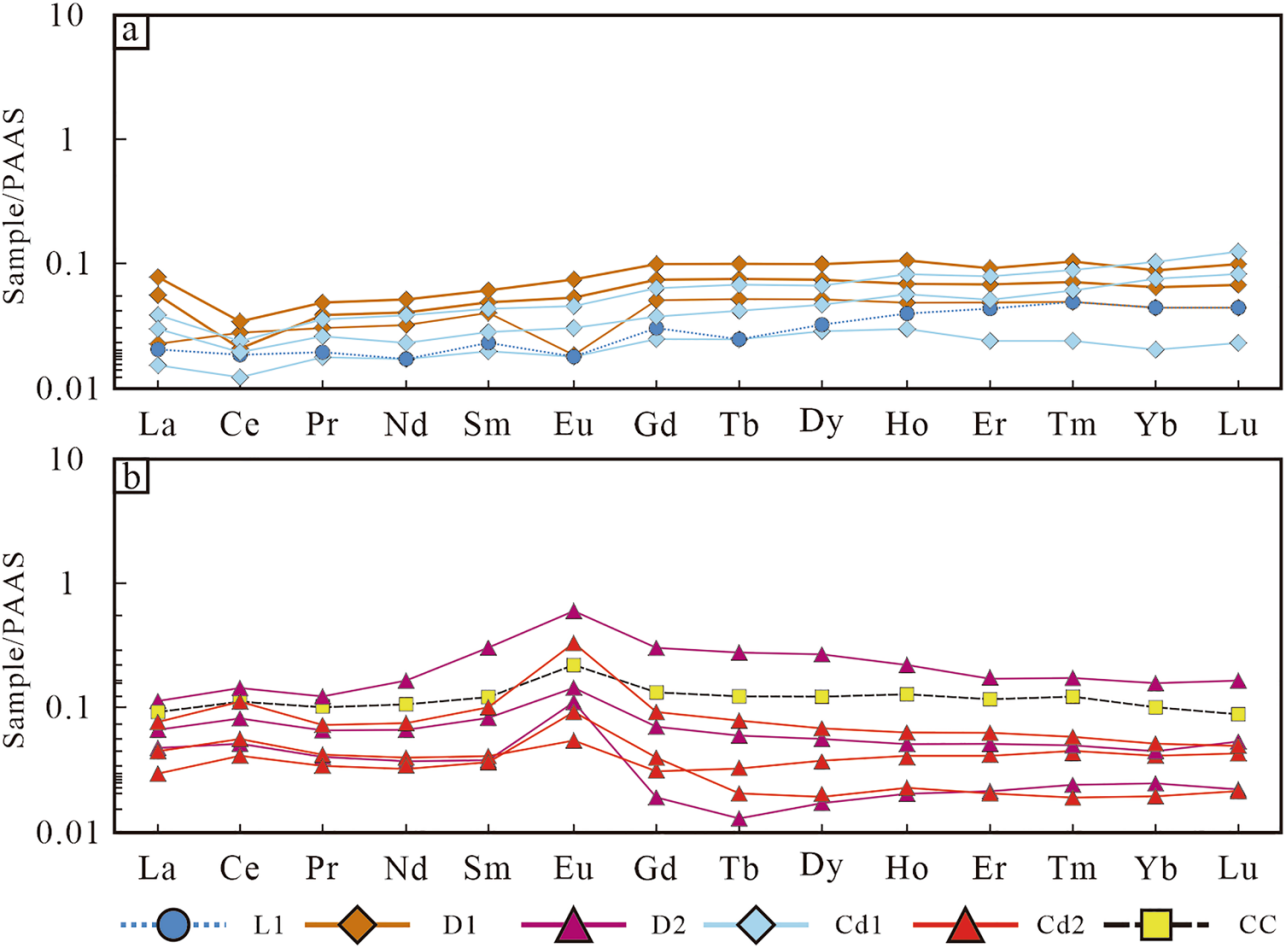
Post-Archean average shale (PAAS)-normalised rare earth element (REE) patterns. (**a**) REE distribution map of limestone and karst-related dolomite, showing relevant characteristics influenced by karstification; (**b**) REE distribution map of CC and hydrothermal-related dolomite, showing relevant characteristics influenced by hydrothermalism. The PAAS data were obtained from McLennan^40^.

The rare earth element (REE) data were normalised to PAAS^40^. L1, D1 and Cd1 had relatively flat REE patterns while heavy rare earth elements (HREEs) were enriched (average Nd_SN_/Yb_SN_ = 0.63, n = 7) with mild negative Eu anomalies and negative Ce anomalies (average δEu = 0.79, n = 7; average δCe = 0.76, n = 7; Fig. 7a). D2, Cd2 and CC presented positive Eu anomalies (average δEu = 2.46, n = 7), mild positive Ce anomalies (average δCe = 1.35, n = 7) and HREE enrichment (average Nd_SN_/Yb_SN_ = 1.4, n = 7) (Table 2, Fig. 7b).

**Table 2:** Rare earth element data of sample rocks in middle Permian in SW Sichuan Basin of WL-Wulong section.

Sample ID	Minerals	Rare Earth Elements (REEs)/(μg/g)																	Nd_N_/Yb_N_
		La	Ce	Pr	Nd	Sm	Eu	Gd	Tb	Dy	Ho	Er	Tm	Yb	Lu	ΣREE	δEu	δCe	
Sp-05B	L1	0.0209	0.0188	0.0193	0.0177	0.0234	0.0185	0.0300	0.0258	0.0321	0.0404	0.0456	0.0494	0.0461	0.0462	0.43	0.69	0.90	0.38
Sp-06-1	D1	0.0576	0.0214	0.0385	0.0413	0.0486	0.0556	0.0773	0.0775	0.0748	0.0706	0.0702	0.0741	0.0674	0.0693	0.84	0.88	0.59	0.61
Sp-06-3	D1	0.0236	0.0276	0.0306	0.0324	0.0414	0.0185	0.0515	0.0517	0.0513	0.0505	0.0491	0.0494	0.0461	0.0462	0.57	0.40	0.96	0.70
Sp-06-2	Cd1	0.0157	0.0136	0.0181	0.0177	0.0198	0.0185	0.0258	0.0258	0.0299	0.0303	0.0246	0.0247	0.0213	0.0231	0.31	0.81	0.73	0.83
Sp-04-1	Cd2	0.0505	0.0788	0.0491	0.0488	0.0490	0.0520	0.0307	0.0329	0.0371	0.0402	0.0411	0.0447	0.0413	0.0431	0.64	1.31	1.60	1.18
Sp-05-1	D2	0.1171	0.1529	0.1423	0.1822	0.3209	0.6019	0.3047	0.2842	0.2692	0.2220	0.1754	0.1728	0.1560	0.1617	3.26	1.92	1.38	1.17
Sp-07	D2	0.0540	0.0602	0.0472	0.0436	0.0434	0.1111	0.0193	0.0129	0.0171	0.0202	0.0211	0.0247	0.0248	0.0231	0.52	3.54	1.18	1.76
Sp-08-1	CC	0.0933	0.1053	0.1027	0.1055	0.1299	0.2596	0.1567	0.1292	0.1282	0.1312	0.1228	0.1235	0.1028	0.0924	1.78	1.81	1.05	1.03
Sp-04-2	D1	0.0908	0.0587	0.0723	0.0738	0.0813	0.0817	0.1100	0.1054	0.0926	0.1023	0.0977	0.1015	0.0946	0.1008	1.26	0.85	0.83	0.78
Sp-04-5a	Cd1	0.0431	0.0396	0.0592	0.0638	0.0669	0.0718	0.0831	0.0846	0.0838	0.0915	0.0915	0.0954	0.1015	0.1085	1.08	0.96	0.72	0.63
Sp-04-5b	Cd1	0.0338	0.0299	0.0485	0.0431	0.0515	0.0538	0.0623	0.0662	0.0708	0.0777	0.0746	0.0815	0.0885	0.0923	0.87	0.95	0.55	0.49
Sp-04-2	Cd2	0.0836	0.0979	0.0796	0.0826	0.0958	0.3425	0.0935	0.0886	0.0801	0.0813	0.0774	0.0715	0.0689	0.0653	1.41	3.62	1.28	1.20
Sp-05-2	D2	0.0766	0.0859	0.0736	0.0756	0.0908	0.2186	0.0796	0.0721	0.0707	0.0685	0.0616	0.0586	0.0465	0.0697	1.15	2.57	1.20	1.63
Sp-04-3	Cd2	0.0389	0.0539	0.0352	0.0405	0.0312	0.0947	0.0459	0.0356	0.0286	0.0365	0.0203	0.0219	0.0223	0.0228	0.53	2.46	1.76	1.82

Ce is extremely sensitive to redox conditions. In an oxidising environment, soluble Ce^3+^ would be oxidised into insoluble Ce^4+^, which could first enter other particles and then precipitate while resulting in negative Ce anomalies. Positive Ce anomalies may be present in dysoxic environments^49^. Positive Eu anomalies in carbonate are generally caused by hydrothermal fluid, river water and other factors^50^. The ionic radius of Eu^3+^ increases upon being reduced to Eu^2+^, which facilitates the replacement of Ca^2+^ and entrance into carbonate lattices. In the study area, L1, D1 and Cd1 generally had mild negative Ce anomalies, indicating that the diagenetic environment of the samples was a relatively open suboxic-oxic environment. This further supported that the top of the Qi2 member had a diagenetic environment to accept karstification. The D2, Cd2 and CC samples had positive Ce and Eu anomalies with relative HREE enrichment^51^, which were the indication of hydrothermalism characteristics.

### Oxygen and carbon isotopes

**Figure 8:**
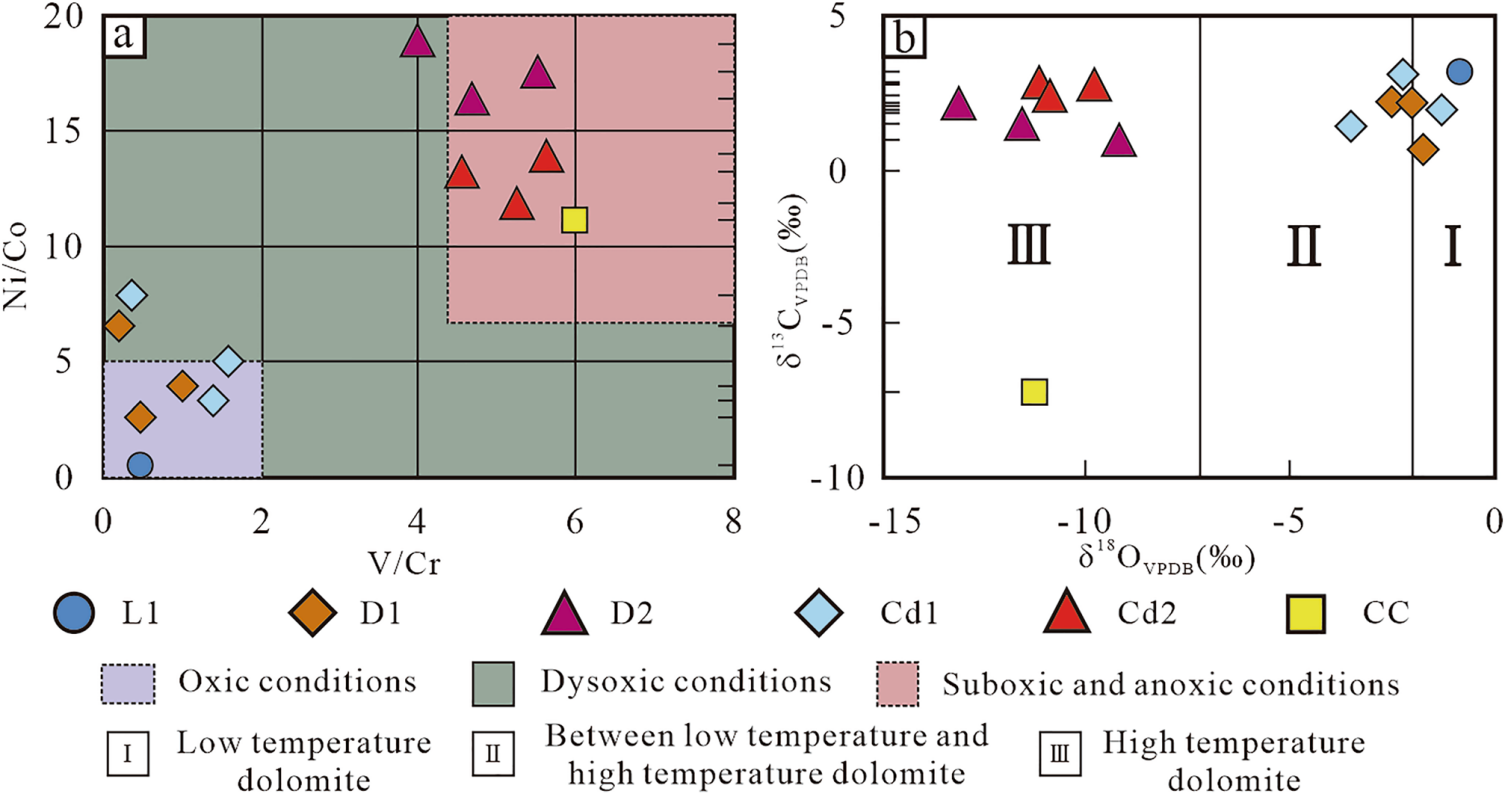
Stable isotope and trace element distribution patterns of sample rocks in the Second Member, Qixia Formation and Wulong section. (**a**) Cross-plot of V/Cr and Ni/Co values. (**b**) Cross-plot of δ^13^C_VPDB_ and δ^18^O_VPDB_ values. Palaeoredox conditions range of trace element values from Bryn and Manning^48^. Typical isotopic values of dolomite formed under low temperatures and high temperatures from Allan^52^.

The δ^13^C_VPDB_ and δ^18^O_VPDB_ values of L1 were 3.44‰ and –1.2‰, respectively. The δ^13^C_VPDB_ and δ^18^O_VPDB_ values of D1 and Cd1 were 0.84–3.28‰ and 3.8–1.9‰, respectively. The δ^13^C_VPDB_ values of D2 and Cd2 ranged from 0.99‰ to 2.78‰ while δ^18^O_VPDB_ ranged from 13.18‰ to –9.18‰. The δ^13^C_VPDB_ and δ^18^O_VPDB_ values of CC were 7.27‰ and 11.31‰, respectively (Table 1, Fig. 8b).

The mean δ^13^C_VPDB_ of D1 and Cd1 (2.01‰, n = 6) was very close to that of the mean of middle Permian marine facies carbonate in the upper Yangtze Platform (2.86‰, n = 78) ^53^. δ^18^O_VPDB_ (3.8‰ to 1.9‰) was similar to that of middle Permian dolomite^54^ while having low-temperature dolomite characteristics^52^. This demonstrated that the formation of D1 and Cd1 might be related to dolomitisation from the shallow burial stage to the penecontemporaneous stage under low temperatures^55,56^. D2 and Cd2 both had relatively low δ^18^O_VPDB_ values. The similar C-O isotopic characteristics confirmed the identical diagenetic fluid composition of D2 and Cd2 and their high-temperature dolomite characteristics^52^. Under near-surface temperature, the δ^18^O_VPDB_ of CC precipitated in atmospheric with higher than − 8‰ of water^57^. The relatively low δ^18^O_VPDB_ value (11.31‰) of CC indicated it might be influenced by the high temperatures from the middle-deep burial stages. It was speculated that the extremely low δ^13^C value (− 7.27‰) might be due to the influence of organic acid invasion on samples during the burial process^58^. From the perspective of carbon and oxygen isotopes, there were obvious products of the penecontemporaneous-shallow burial stage and the middle-deep burial stage in the study area.

## Discussion

### Paragenesis

**Figure 9:**
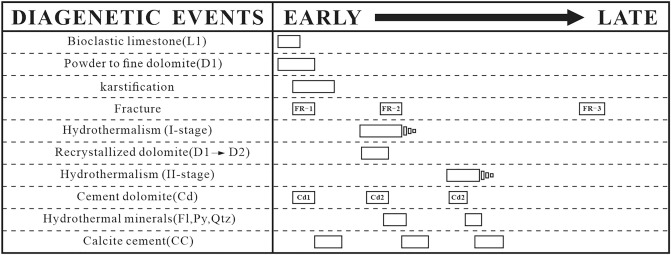
Diagenetic sequence of middle Permian Qixia Formation (Qi2) in SW Sichuan Basin.

Carbonate platform sediments were formed in the middle Permian Qixia Formation in the southwestern region of the Sichuan Basin. The Qi2 member mainly belongs to the particle beach facies sediments^59^. Multi-cycle superposition sequences of thin-middle layered dark grey bioclastic limestone and thick-layered light grey crystalline dolomite were present vertically. The entire sedimentation process of the Qi2 member involved a gradual decline in the relative sea level with a short-term rapid increase in the relative sea level. Once the sedimentation was complete, the Qi2 member experienced tectonic uplift movement^60^ while exposing the sedimentary strata. These factors provided ideal conditions for the development of karst. Therefore, multiple dolomite facies were developed in the Qixia Formation due to the complicated burial process. A paragenetic sequence was established as shown in Fig. 9.

The paragenetic sequence (Fig. 9) was established based on the cross-cutting relationships obtained from the field and the thin sections. L1 was the earliest original rock (Fig. 4a) while D1 recrystallised from C1 during the penecontemporaneous period (Fig. 4b). In this stage, the strata experienced Eogenetic karstification and Cd1 was transported into karst channels by fluid media. Once the sedimentation was complete, the stratum was uplifted to the surface and exposed by the geotectonic movement while causing Telogenetic karstification. Some Cd1 was filled with karst channels. Under the influence of matrix activation in the middle and late diagenetic stages^36^, hydrothermal fluid entered the strata along the faults, causing D1 to recrystallise into medium-coarse crystalline dolomite (D2). Late dolomite cement (Cd2) rapidly got filled with cracks (Fig. 4c,d). Hydrothermal minerals (e.g., Py and Fl) filled the intercrystal pores of Cd2 and its formation was slightly later than that of Cd2. Calcite cement (CC) in microfractures intersected the hydrothermal minerals and Cd2 (Fig. 4c,d).

### Karstification

There were typical karstification signs at the top of the Qi2 member such as lithologic mutation surfaces caused by sea level changes^44^ and karst channels with irregular morphology^45^ (Fig. 2). This karst system played a significant role in creating spaces for reservoirs and providing pathways for the migration of hydrothermal fluid. However, the formation of the karst system was controlled significantly by the karstification intensity, which was closely related to the vertical sequence of cycles. Karst water at the top of the sedimentary cycles could migrate along strata positions with high initial porosity and permeability. In the early stages of karstification, highly unsaturated karst water may have diffused through interparticle pores among loose limestone while forming solution pipes and vugs for fluid conduction. With the continuous migration of karst water, its dissolving capacity decreased due to the difference in structural selection and saturation. Therefore, the karst water could no longer form new solution pipes and could only diffuse via existing solution pipes. When the karst water supply increased (for example, rainfall increased), it might have exceeded the seepage capacity of the solution pipes. As a result, karst water seeped out via interparticle pores or fractures out of solution pipes, which further expanded into existing solution pipes and vugs to form a ‘karst channel-pore’ system^61^. Karst channels were found to be the result of an expanded solution.

**Figure 10:**
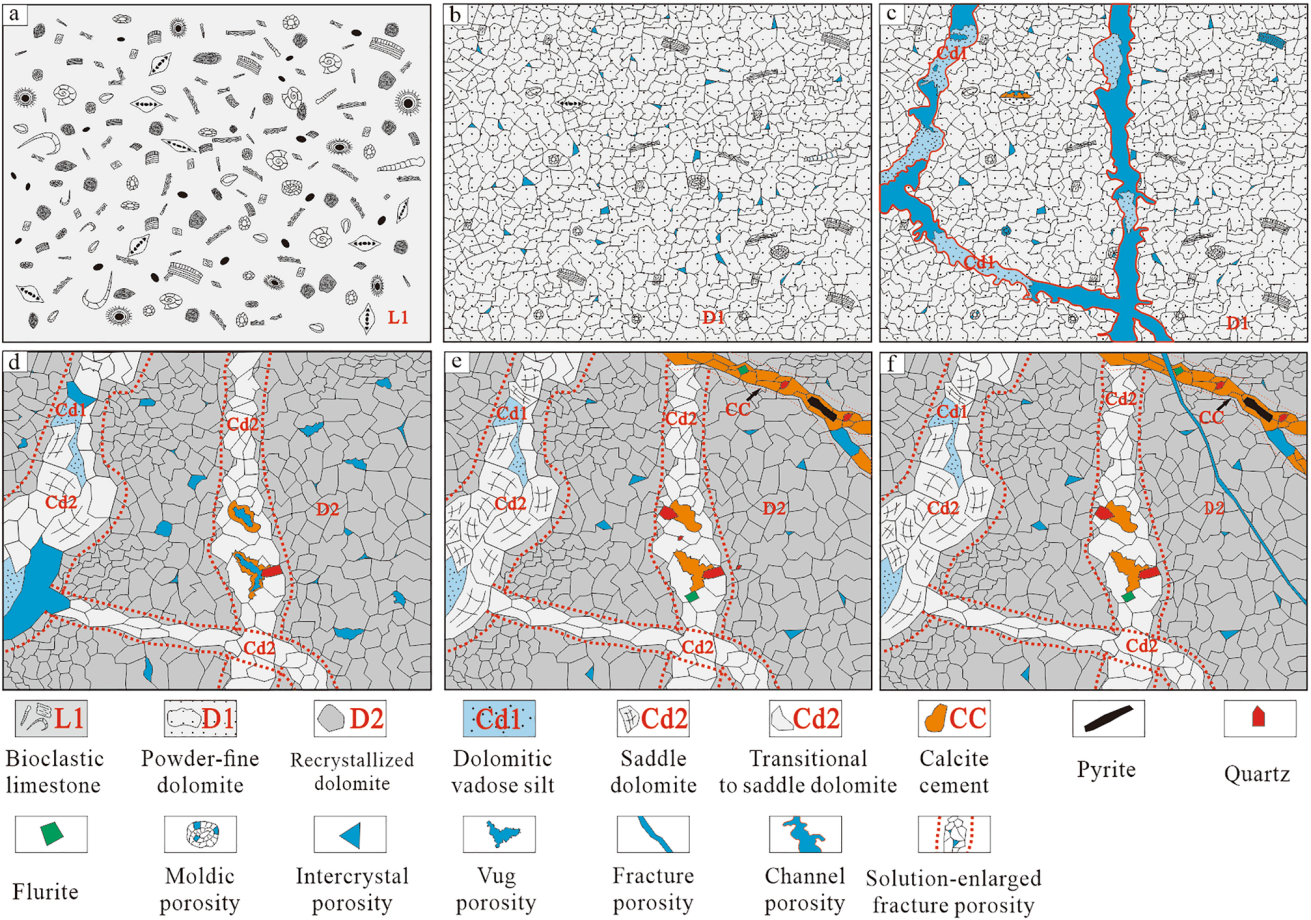
Alteration pattern of karstification and hydrothermalism on dolomite reservoir in the Second Member, Qixia Formation and Wulong section. (**a**) Undolomitised stage. (**b**) Penecontemporaneous dolomitisation stage. (**c**) Karstification stage. (**d**) I-stage hydrothermalism. (**e**) II-stage hydrothermalism. (**f**) Late tectonic movement stage.

The formation of the karst system was accompanied by sea level rising and dolomitisation in limestone strata under the collective action of active and latent reflux. Discontinuous dolomite strata formed, resulting in the alternating development of dolomite and undolomitised limestone strata in the area^62,63^. Therefore, the loose limestone strata (Fig. 10a) were replaced by powder and fine dolostone (Fig. 10b) due to penecontemporaneous dolomitisation. The geochemical characteristics of samples also proved the suboxic-oxic environmental conditions of the shallow burial stage. Additionally, Cd1 was transported with karst water and entered karst channels and filled them (Fig. 10c).

### Hydrothermalism

The strong Indosinian movement reactivated the major basement fault in the study area^36^. According to previous studies, a relatively high palaeo-heat flow value is present in the southwestern region of the Sichuan Basin^64^. The hydrothermal phenomenon is a manifestation of the thermal effect of magmatic activity. Deep Mg^2+^-rich fluid influenced by the high temperature, entered the Qixia Formation along the major Indosinian Fault (base fault in the Wulong Section; Figs. 1b and 3b). The small tectonic fractures accompanied the formation of the fault and the early karst system provided favourable pathways for the migration of deep hydrothermal fluids^65,66^. These tectonic fractures were mainly distributed in dolomite strata in dendritic form. Dolomitisation was not discovered in relatively compact limestone strata and only a few fractures were developed and filled with CC (Fig. 4a). After hydrothermal fluid entered the dolomite strata, D1 was transformed locally by FR-1 and recrystallised into middle-coarse crystalline dolomite (D2). Some of the hydrothermal fluid that entered the karst system might have resulted in the solution-enlarged pores that developed as karst channel and the recrystallisation of vadose silt dolomite (Cd1) into middle crystalline dolomite (Cd2, transition form of saddle dolomite). Furthermore, Cd2 was precipitated directly in some pores (Fig. 10d). After Phase-I hydrothermalism, the recrystallised dolomite strata might have undergone Phase-II hydrothermalism alteration due to the influence of many factors such as base activation. The Phase-II hydrothermal Mg^2+^-rich fluid resulted in the recrystallisation of the middle crystalline dolomite (Cd2, transition form of saddle dolomite) in the karst system into coarse crystalline dolomite (Cd2, transition form of saddle dolomite) or saddle dolomite (Cd2). When Mg^2+^ in the hydrothermal fluid was consumed, residual pores in fractures would have been filled with hydrothermal minerals and CC (Fig. 10e). Once the hydrothermal activity ceased, the reconstructed strata were influenced by tectonic movements in the Yanshanian or Himalayan Periods while generating a series of unfilled tectonic fractures (Fig. 10f).

Intercrystal porosity was developed in thick-massive dolomite strata with the largest porosity of 8% (Fig. 4b). Intercrystal porosity was well preserved, which could indicate changes in the solution pipes. Under the influence of Phase-I hydrothermalism, the protolith was recrystallised (compacted with few residual intercrystal porosities). After recrystallisation, the relatively compact crystalline dolomite strata restricted the diffusion of deep hydrothermal fluid to some extent. Consequently, it was difficult for the late hydrothermal fluid to continuously fill previous residual intercrystal porosity. However, it could only migrate along residual fractures. Hydrothermal fluids dissolved and precipitated simultaneously, forming a series of harbour-shaped corrosion rims and CC.

The reservoir space in the thin dolomite strata was barely developed and the porosity was found to be 1% (Fig. 4d). This could imply that the thin-layered dolomite was more influenced by the late hydrothermal alteration than the thick-massive dolomite strata. Additionally, the loose karst system had more favourable solution pipes than the surrounding matrix rocks having broken fractures. When deep hydrothermal fluid entered the Qixia Formation strata along the basement fault, it might have first filled the karst system due to its relatively good porosity and permeability, solution channels and fluid reservoir space. Consequently, the karst system was completely filled and only a few pores were formed. Therefore, hydrothermalism in the study area was dominated by the filling effect. Intercrystal porosity in dolomite was first formed during penecontemporaneous dolomitisation. Pores were further shrunk due to hydrothermal recrystallisation of matrix dolomite while subsequently forming residual pores transformed by hydrothermal fluid.

### Regional comparisons and implications

In order to establish how representative, the data from the South China study area are, it is useful to compare them to other known karstification (hydrothermalism) -controlled dolomite reservoirs. These cases are distributed in the southern Sichuan Basin of China, offshore Spain and western Canada. Alteration mechanisms and controlling factors have been described in detail. However, the relationship between these mechanisms has not been systematically established.

**Figure 11:**
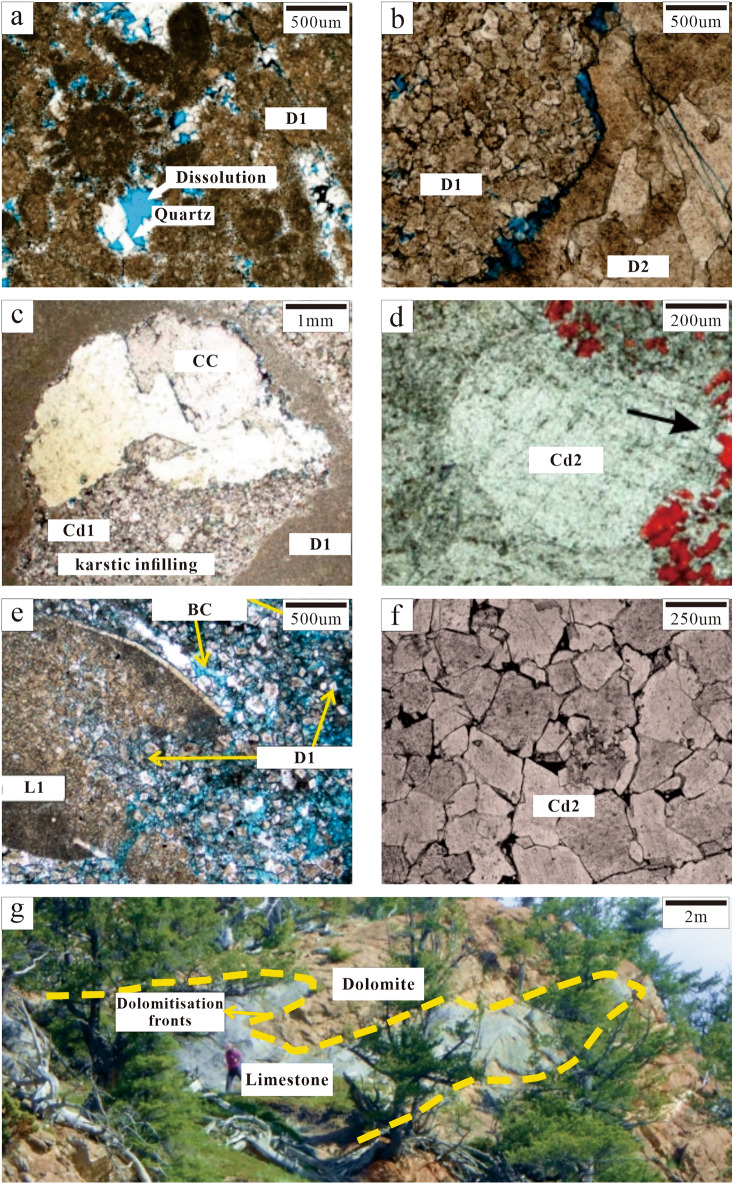
Alteration effects of karstification and hydrothermalism in south China, offshore Spain and western Canada. These photos are respectively cited from Zheng^67^, Koeshidayatullah^68^ and Rodríguez-Morillas^69^. (**a**) Micritic dolomite (D1) with residual particles mimics replacement and later filled with quartz cements. southwestern Sichuan Basin, south China^67^. (**b**) Euhedral to subhedral fine-grained dolomite (D1) and medium-coarse dolomite (D2) with curved crystals. southwestern Sichuan Basin, south China^67^. (**c**) Vug porosity partially filled by fine-coarse dolomite (Cd1) and calcite cement (CC). Valencia Trough, offshore Spain, western Mediterranean Sea^69^. (d)Calcitization of saddle dolomite (Cd2). Valencia Trough, offshore Spain, western Mediterranean Sea^69^. (**e**) Partially replaced of micritic limestones (L1) by euhedral-subhedral dolomites (D1) in the halo zones, intercrystal porosity (BC) developed and porosity was 11%. western Canada^68^. (**f**) A typical saddle dolomite (Cd2) in core with typical of euhedral fabric and coarser crystal size, porosity was less than 1%. western Canada^68^. (**g**) Termination of dolomite bodies (brown) into the limestone (grey), showing a scalloped-shaped front. western Canada^68^.

Similarly located in the southwest of the Sichuan Basin, southeast of this study area, four types of middle Permian dolomites have been revealed in the field (Fig. 11a,b). Petrographic and geochemistry studies have described the fabric and geochemical characteristics of the four types of dolomites as well as the dolomitisation involved in their evolution process (seepage/reflux and hydrothermal dolomitization). It indicated that the distance between the dolomitisation process and fault zones might be related to the distribution of different phases of dolomite^67^. This situation is similar to what has been that observed in the Wulong section. However, it is still not understood how the porosity evolved.

Porosity evolution has been described from the well-exposed, partially dolomitized Cambrian carbonate platform of the Mount Whyte Formation and Whirlpool Point locality of western Canada (Fig. 11g). The dolomitisation fronts and halo zones have higher porosity than their core, which is associated with the progressive decrease in porosity inboard of the reaction front (Fig. 11e,f), caused by the increase in crystal size, and decrease in pore size during recrystallization in the core of the body^68^. The porosity evolution involved in this study is only explained from the perspective of genesis while the example of western Canada illustrates why porosity increases or decreases from the perspective of mechanism, which supports the research.

Compared to the south China data and Cambrian dolomite reservoirs of western Canada, the phenomenon described by the Spanish case has a better similarity with this study. Currently, dolomitized upper Jurassic limestones constitute the main reservoir of the Casablanca oil field, located in the western Mediterranean Sea. The dolomite reservoir was also affected by the alteration effects of karstification and hydrothermalism (Fig. 11c,d). It had two distinct characteristics: (i) the reservoir rock was uplifted and subaerially exposed between the Paleogene compression and the Neogene extension while significantly increasing their porosity; (ii) the porosity was obliterated by successive cement precipitations^69^. The outcomes were similar to the phenomenon investigated in South China having multistage hydrothermal fluids resulting in multiple recrystallizations of dolomite and a decrease in porosity.

The similarity in fabric development and paragenesis of global compound alteration effects of karstification and hydrothermalism was significant. This may imply a uniform carbonate rock alteration mechanism, regardless of geological age. This study attempted to establish the relationship between complex carbonate rock alteration and reservoir porosity evolution while emphasizing porous conditions in the karst system than the surrounding matrix. However, the preservation of porous conditions under fracture networks and hydrothermalism was challenging. Furthermore, the preservation of porous conditions was also influenced by the thickness of the reservoir. The intercrystal porosity of the thick-massive reservoir was well preserved after early dolomitization and hydrothermal alteration (Figs. 2a and 4b). In contrast, the thin reservoir was more susceptible to late hydrothermal alteration than the thick-massive reservoir, which resulted in a decrease in porosity, the obvious filling of fractures as well as intense compaction of the reservoir (Fig. 4a,c). Therefore, the alteration effect of karst and hydrothermal fluid was mainly destructive to carbonate in the study area. Differences in spatiotemporal distribution in local porosity were controlled by the connectivity of solution pipes and the reservoir thickness.

## Conclusions

This study included comprehensive research on the petrology and geochemistry of the middle Permian Qixia Formation in the Wulong Section in the southwest region of the Sichuan Basin. The field observations, petrological and geochemical analyses revealed multiple dolomite facies. The diagenetic sequence was found to result from the alternation of karstification and hydrothermalism effects on the carbonate strata.

- The different dolomite facies in the middle Permian Qixia Formation in the Wulong Section in the southwest region of the Sichuan Basin represented different redox environments. According to geochemical data, D1 and Cd1 were related to karstification and had the following characteristics: enriched δ^18^O_VPDB_ values, negative Ce anomalies and trace elements of oxic environments. D2 and Cd2 were related to hydrothermalism and had the following characteristics: depleted δ^18^O_VPDB_ values, positive Eu anomalies and trace elements of dysoxic environments.
- The fault zone and karst system created pathways for the entrance and migration of hydrothermal fluid. The fault zone provided strata with basement faults and multiphase fractures. The karst system contained numerous irregular channels and vugs under the exposed surface, which provided pathways for the entrance of hydrothermal fluid. Hydrothermal fluid entered from the basement fault into the Qixia Formation and migrated along the favorable pathways (e.g. karst system and fracture network). Hydrothermal fluid can migrate readily through the loose early karst system along the fracture network. This migration pattern revealed the heterogeneity of reservoir alteration.
- The combined effects of karstification and hydrothermalism mainly resulted in the degradation of the dolomite reservoirs in the Qixia Formation of the Wulong Section. Although karstification provided a basic porosity framework for the dolomite reservoir, the karstification-filling effect of multiphase hydrothermalism was detrimental to the karst reservoirs. The local residual intercrystal porosity in the matrix dolomite was caused by different connectivity among pathways in the same strata sequence. The thickness of the reservoir may also have impacted the integrity of the reservoir spaces. Following the same diagenetic alteration, the thick-massive dolomite in the region could preserve reservoir spaces better than the thin-layered dolomite.

## Data Availability

The datasets used and analyzed during the current study are available from the corresponding author on reasonable request.
